# Supplementary material for: ‘She convinced me’- partner involvement in choosing a high risk birth setting against medical advice in the Netherlands: A qualitative analysis
Source: PLoS One. 2020 Feb 20;15(2):e0229069. doi: 10.1371/journal.pone.0229069 (PMC7032726; doi:10.1371/journal.pone.0229069)
Supplement: S1 File — (DOCX) [file pone.0229069.s001.docx]

**Keywords**

unassisted childbirth, home birth, risk, freebirth, care provision, partners, midwives, obstetricians, couples, choices

**List of abbreviations**

UC: unassisted childbirth
WONDER (study): Why women want Other or No DElivery caRe
VBAC: vaginal birth after cesarean
PPH: Post Partum Hemorrhage > 1000 ml in previous delivery
MPV: history of Manual Placental Removal
