# Supplementary material for: ‘She convinced me’- partner involvement in choosing a high risk birth setting against medical advice in the Netherlands: A qualitative analysis
Source: PLoS One. 2020 Feb 20;15(2):e0229069. doi: 10.1371/journal.pone.0229069 (PMC7032726; doi:10.1371/journal.pone.0229069)
Supplement: Attachment — Submitted filename: renamed_4a2a2.docx [file pone.0229069.s002.docx]

**Review of the paper entitled « ‘She convinced me’- Partner involvement in choosing a high risk birth setting against medical advice in the Netherlands: a qualitative analysis. » by Martine Hollander et al.**

I have read with great interest the paper of M. Hollander et al. and I think it is of scientific quality high enough and deserves publication in PLOS, providing the authors are willing to give some precisions.

In summary, the authors conducted a qualitative research in order to understand better the role of women’ partners choosing high risk birth setting against medical advice. They interviewed 21 subjects and found 4 main themes: “Talking it through”, “A shared vision”, “Defending our views”, “Doing it together”. The overarching theme that covered all the others was “she convinced me”.

First, I would like to congratulate the authors for their hard work putting through so many interviews and for the quality of their transcriptions.

In general, the manuscript is well written and easy to read. However, it is a little bit long and the authors should go forward in their discussion and not only rephrase most of the results.

A weakness of this study also lies in its retrospective nature and sometimes the long delay between pregnancy / delivery and the interview. This could have introduced a recognition bias. Furthermore, can the authors precise if the couples had subsequent pregnancies and their choices for delivery / issue of the pregnancy?

The fact that the authors interviewed the partners of women previously interviewed in another study could also have introduced a bias as they might have read the results of the previous study. Obviously, this is also a strength of this study since it helped increasing the number of partners interviewed.

Theses limits should appear clearly in their discussion.

By any chance, did the authors observed if the partner answers were different when the woman was / was not here? Did they have a chance to transcript some of the physical language not otherwise expressed?

When reading the study, I can’t help wonder whether the way these couple are dealing with the choice of delivery is unique or are they reproducing a pattern they are used to for different fields? Like buying a house, choosing where to live, work, etc etc….

It would have been great to submit a general questionnaire to the couples to understand the way they usually process decisions and see whether their choice for birth place is different or not.
